# Signatures of circulating microRNA in four sarcoma subtypes

**DOI:** 10.7150/jca.34723

**Published:** 2020-01-01

**Authors:** Hanna Kosela-Paterczyk, Agnieszka Paziewska, Maria Kulecka, Aneta Balabas, Anna Kluska, Michalina Dabrowska, Magdalena Piatkowska, Natalia Zeber-Lubecka, Filip Ambrozkiewicz, Jakub Karczmarski, Michal Mikula, Piotr Rutkowski, Jerzy Ostrowski

**Affiliations:** 1 Department of Soft Tissue, Bone Sarcoma and Melanoma, Maria Sklodowska-Curie Institute - Oncology Centre, Warsaw, Poland; 2 Department of Gastroenterology, Hepatology and Clinical Oncology, Centre of Postgraduate Medical Education, Warsaw, Poland; 3 Department of Genetics, Maria Sklodowska-Curie Institute - Oncology Centre; 02-781 Warsaw, Poland

**Keywords:** miRNA, sarcoma, diagnosis, next generation sequencing

## Abstract

**Background**: Sarcomas are rare malignant tumors of mesenchymal origin. The discovery of circulating biomarkers with high diagnostic value could supplement diagnosis of this heterogenous group of tumors. The aim of this study was to identify the profiles of circulating miRNA (c-miRNAs) in four groups of common bone and soft tissue sarcomas.

**Methods**: At the time of diagnosis, blood samples were collected from 86 patients: 36 with locally advanced/unresectable/metastatic gastrointestinal stromal tumor (GIST) who received first-line treatment with imatinib; 16 with locally advanced osteosarcoma (OS); 26 with locally advanced synovial sarcoma (SS); and eight with locally advanced Ewing sarcoma (ES). In addition, samples were collected from 30 healthy controls. C-miRNAs were isolated using a miRCURY RNA Isolation Kit, followed by preparation of cDNA libraries and sequencing on the Ion Proton platform.

**Results**: Pair-wise comparisons identified 156 unique c-miRNAs (adjusted P-value < 0.05) showing significant dysregulation between controls and patients; of these, 24, 36, 42, and 99 differentiated controls from pretherapeutic OS, SS, ES, and GIST, respectively. Ten c-miRNAs were commonly altered in at least three sarcoma types. Receiver operating characteristic curves and area under the curve (ROC-AUC) analyses revealed that a four-miRNA diagnostic classifier was able to differentiate controls from ES, GIST, OS, and SS, with AUC-ROC values of 1, 0.97, 0.95, and 0.94, respectively.

**Conclusions**: Aberrant miRNA expression signatures were identified in serum from patients with four different sarcoma subtypes. Differences in miRNA expression profiles between sarcoma patients and healthy volunteers suggest that miRNAs may play a role in sarcoma development.

## Background

Sarcomas, rare tumors of mesenchymal origin, account for approximately 1% of all adult cancers. Soft tissue sarcomas (STSs) comprise at least 60 histological subtypes, and even more molecular subsets. For example, there are at least six major histological types of bone sarcoma. The majority (close to 75%) of sarcomas arise from soft tissue, with an additional 15% being gastrointestinal stromal tumors (GISTs) and 10% being bone sarcomas (BSs) 1. The primary treatment for sarcomas is surgery, although (neo)adjuvant chemotherapy is the main treatment for some BS cases 2. The use of perioperative chemotherapy for STS is uncommon; however, up to date guidelines state that it should be an option for high-risk individuals; this is because approximately half of all STS patients with intermediate or high-grade tumors develop metastatic disease, and the 5 year overall survival is approximately 55% 1,3. Increased understanding of the molecular mechanisms underlying GIST has led to introduction of targeted therapy with receptor kinase inhibitors. Imatinib mesylate was the first effective systemic therapy for GIST; this treatment shows high efficacy in advanced cases and has improved the outcomes of patients markedly 4. Making a diagnosis of sarcoma is complicated and is based on adequate imaging and pathological examination, often aided by molecular/cytogenetic diagnostics. As a supplement to traditional diagnostic methods, identifying circulating biomarkers with high diagnostic utility is essential.

Non-coding microRNAs (miRNAs) affect cellular processes by regulating gene expression. As such, over-abundance may play a role in pathogenesis of various diseases, including human neoplastic disorders. It is assumed that cell-free circulating miRNAs are potential cancer biomarkers because they are stable molecules that show expression profiles that correlate specifically with different types of cancer; in addition, they are sampled easily in a relatively non-invasive manner and are detected readily by various techniques 5.

Lawrie et al. were the first to identify circulating miRNAs in serum and to suggest their potential as cancer markers 6. They found that serum miRNAs (including miR-155, miR-210, and miR-21) in the serum of diffuse large B-cell lymphoma patients were higher than those in in healthy individuals. More interestingly, high miR-21 levels in these patients were associated with relapse-free survival. Mitchell and co-workers 7 found that circulating miRNAs are remarkably stable and are protected from endogenous RNase activity. Following these observations, several studies reported miRNAs differentially expressed in serum or plasma from patients with gastric cancer, non-small-cell lung cancer, hepatocellular carcinoma, rhabdomyosarcoma, head and neck squamous cell carcinoma, breast cancer, bladder cancer, colorectal cancer, epithelial ovarian cancer, renal cancer, pancreatic cancer, esophageal squamous cell carcinoma, chronic lymphocytic leukemia, melanoma, and Hodgkin lymphoma 8-18.

The aim of this study was to identify circulating miRNA (c-miRNA) profiles in serum or plasma from four groups of individuals with common bone and soft tissue sarcomas: osteosarcoma (OS), synovial sarcoma (SS), Ewing sarcoma (ES), and gastrointestinal stromal tumor.

## Methods

### Patients

Selection of patients and controls was performed at the Department of Soft Tissue/Bone Sarcoma and Melanoma, Maria Sklodowska-Curie Institute - Oncology Center, Warsaw, Poland, which is the reference center for adult sarcomas and is integrated into the European Reference Network EURACAN. Patients were selected according to the following criteria: 1) categorized into homogenous groups of sarcoma patients; and 2) they had a well-established pathological diagnosis performed by expert pathologists specialized in sarcoma diagnostics and confirmed by molecular testing (i.e., cytogenetics results showing translocation of t(X;18) and t(11;22), which is characteristic of SSs and ESs, respectively, and genotyping revealing the presence of oncogenic *KIT* and *PDGFRA* driver mutations in GISTs). Computed tomography (CT) or magnetic resonance imaging (MRI) was performed to establish disease staging. The study was approved by the local Bio-Ethics Committee and was conducted in accordance with Good Clinical Practice Guidelines (decision 13/2008). All patients provided written informed consent to participate.

The following patients were included in the analyzed groups: 1) Patients with locally advanced/ unresectable/metastatic GIST who received first-line treatment with imatinib (400 mg OD) until disease progression or unacceptable toxicity, with response to treatment assessed by CT scan every 2-3 months; 2) An OS group comprising patients with localized tumors treated with preoperative chemotherapy (doxorubicin and cisplatin) and 3-6 cycles of post-operative chemotherapy consistent with the same as preoperative scheme; 3) SS patients treated with the internal extensive combined modality treatment protocol, which comprised two cycles of high-dose ifosfamide, preoperative radiotherapy, radical surgery, and post-operative chemotherapy (two cycles of high-dose ifosfamide and two cycles of doxorubicin and cisplatin); and 4) ES patients treated with chemotherapy comprising multi-drug anthracycline- and ifosfamide-based chemotherapy prior to local treatment (surgery and/or radical radiotherapy), followed by continuation of chemotherapy (treatment as a whole lasted 12 months). The characteristics of the four patient groups and controls are listed in Table 1.

### Blood sample collection

Blood samples were obtained by venipuncture using Serum Gel S/7.5 ml collection tubes (Sarstedt S-Monovette), allowed to clot for 60 min at room temperature, and then centrifuged at 1300 ×g for 10 min at 4°C. Next, 500 µl of serum was aliquoted into 1.5 ml siliconized polypropylene microtubes (Sigma-Aldrich, T4816) and stored at -80°C until further use. MiRNAs were isolated from 1200 μl of serum mirVana Paris RNA and Native Protein Purification Kit (Thermo Fisher Scientific) according to the manufacturer's instructions. The quantity of miRNA was checked using the MyQubit microRNA Assay (Thermo Fisher Scientific). The small RNA fraction was detected using the Small RNA Kit (Agilent) and a Bioanalyzer 2100. Samples that passed the quality check were kept at -80°C until further analyses.

### NGS library preparation, sequencing, and data analyses

200ng miRNA was used to construct a sequencing library with an Ion Total RNA‐Seq Kit v2 for Small RNA Libraries and Ion Xpress™ RNA‐Seq Barcode Kit (Thermo Fisher Scientific), according to the manufacturer's protocol. The amount of amplified library and size distribution was assessed on the Bioanalyzer 2100 using a High Sensitivity DNA Kit; the size range for barcoded libraries of miRNA ligation products was 94‐114 bp. The template preparation procedure for clonal amplification of up to 16 miRNA libraries at a concentration of 40 pM, and loading of the Ion PI Chip v3, were performed using The Ion Chef instrument, with a set of reagents from the Ion PI™ Hi-Q™ Chef Kit (Thermo Fisher Scientific). Sequencing was achieved using the Ion Torrent Proton sequencer (Thermo Fisher Scientific) with 500 run flows.

Unmapped bam files were converted into fastq files using a bamToFastq script from bedtools 19. Read mapping to the human genome (hg19), quantification of known miRNAs (according to miRBase release 18 20), and prediction of novel miRNAs was performed using miRDeep2 version 2.0.0.7 21. Differential expression of miRNAs was analyzed using edgeR version 3.20.6 22, using likelihood ratio test. Post-hoc power analysis was conducted with RNASeqPower R package version 1.18.

### Statistical analyses

AUC-ROC values were calculated using R in the pROC package version 1.10 23. Model variable selection and optimization were conducted using the R package glmulti (Vincent Calcagno (2013); glmulti: Model selection and multi-model inference made easy. R package version 1.0.7. https://CRAN.R-project.org/package=glmulti). Functional analysis was conducted using mirPath version 3 24, using gene union. P-values were corrected for testing of multiple hypotheses with Benjamini-Hochberg method. Adjusted p-values less or equal to 0.05 were considered significant.

### Availability of data

The sequencing datasets generated during the current study are available in the European Nucleotide Archive repository under the PRJEB30542 identifier.

## Results

C-miRNA profiles were analyzed by deep sequencing of miRNA transcriptomes isolated from serum obtained from 16, 30, 8, and 36 patients with OS, SS, ES, and GIST, respectively; all were collected at the time of diagnosis. In addition, 30 samples from healthy controls were assessed. There was a significant difference (p-value < 0.05) in age of GIST and SS patients when compared to control group. In 5, 15, and 12 patients with OS, SS, and GIST, respectively, a second blood sample was also collected after treatment. On average, 733051 reads mapped to miRBase were obtained per library, representing 61% of total reads. Altogether, 1419 mature miRNAs were detected, of which 305 generated ten reads on average. Post-hoc power analysis revealed that at power of 0.9,confidence level of 0.05 and coverage of minimum 5x, the minimum detectable fold-change range between 1.6 (GIST group) and 2.1 (ES).

**Figure 1:**
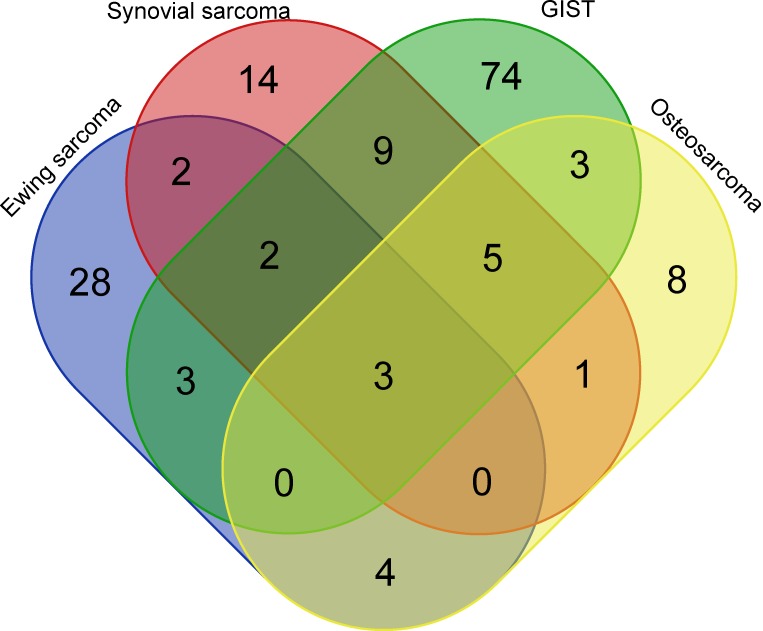
Venn diagram with c-miRNA numbers differentiating sarcoma groups from healthy controls. GIST; gastrointestinal stromal tumor.

Pair-wise comparisons identified 156 c-miRNAs (adjusted P-value < 0.05) showing significant dysregulation between controls and patients, of which 24, 36, 42, and 99 differentiated controls from pretherapeutic OS, SS, ES, and GIST, respectively; log-fold changes ranged from -5.1 to 6.4, -4.0 to 6.0, -7.6 to 7.2, and -2.5 to 7.2, respectively. Interestingly, three (hsa-miR-483-5p, hsa-miR-96-5p and hsa-miR-150-3p) and seven dysregulated miRNAs (hsa-miR-4772-5p, hsa-miR-582-5p, hsa-miR-450b-5p, hsa-miR-124-3p, and hsa-miR-492, hsa-miR-486-5p and hsa-miR-375) differentiated all and at least three tumor types from healthy controls., respectively. (Figure 1). Of note, another studies 25-45 pointed out 11, 5, 21, and 14 of these c-miRNAs as dysregulated among 291, 58, 150, and 68 c-miRNAs identified in OS, SS, ES, and GIST patients, respectively (Tables S1 and S2). Functional analysis revealed that these miRNAs played roles in regulating 50 pathways, including the p53 signaling pathway (hsa-miR-124-3p, hsa-miR-96-5p, hsa-miR-582-5p, hsa-miR-450b-5p, hsa-miR-375, hsa-miR-483-5p, hsa-miR-486-5p and miR-150-3p) and the TNF signaling pathway (hsa-miR-124-3p, hsa-miR-375, hsa-miR-486-5p, hsa-miR-96-5p, hsa-miR-582-5p, hsa-miR-450b-5p, and miR-150-3p; Table S3).

The diagnostic potential of dysregulated pre-therapeutic c-miRNAs was assessed using ROC curve and AUC analyses. Five (hsa-miR-4772-5p, hsa-miR-582-5p, hsa-miR-424-5p, hsa-miR-223-3p, and hsa-miR-106b-3p), nine (hsa-miR-96-5p, hsa-miR-4746-5p, hsa-miR-424-5p, hsa-miR-323a-3p, hsa-miR-375, hsa-miR-223-3p, hsa-miR-133a, hsa-miR-3173-3p, and hsa-miR-9-5p), and four (hsa-miR-582-5p, hsa-miR-150-5p, hsa-miR-450b-5p, and hsa-miR-450a-5p) c-miRNAs in pre-therapeutic OS, ES, and GIST patients, respectively, showed moderate (good) discriminatory power (AUC-ROC values between 0.8 and 0.9). Only two (hsa-miR-142-3p and hsa-miR-9-3p) miRNAs (both in the ES group) showed high (excellent) discriminatory power (AUC-ROC values > 0.9) (Additional file 1 - Table S1). To determine whether the diagnostic ability of a miRNA signature is higher than that of a single miRNA, we also calculated AUC-ROC values for combinations of the miRNAs showing the best discriminatory power for each tumor type. Using linear models of normalized expression values and a stepwise inclusion approach, we identified four-miRNA diagnostic classifiers that differentiated controls from ES (hsa-miR-424-5p, hsa-miR-3173-3p, hsa-miR-142-3p, and hsa-miR-4746-5p), GIST (hsa-miR-151b, hsa-miR-31-5p, hsa-miR-345-5p, and hsa-miR-486-5p), OS (hsa-miR-133a, hsa-miR-223-3p, hsa-miR-450b-5p, and hsa-miR-548q), and SS (hsa-miR-3613-3p, hsa-miR-450b-5p, hsa-miR-486-5p, and hsa-miR-532-5p) with high discriminatory power (AUC-ROC = 1, 0.97, 0.95, and 0.94, respectively).

Several c-miRNAs differentiated pre-therapeutic patients with particular types of tumor (Table 2 and Table S4). The greatest number of miRNAs differentiated GIST from ES (164 c-miRNAs) and GIST from OS (103 c-miRNAs); other pair-wise comparisons identified smaller numbers of tumor-differentiating c-miRNAs.

In five patients with OS, post-therapeutic c-miRNA profiles identified one c-miRNA (hsa-miR-1307-5p, AUC-ROC = 0.83) whose expression differed significantly between patients and healthy controls, and eight c-miRNAs (hsa-miR-24-3p, hsa-miR-223-3p, hsa-miR-487a, hsa-miR-1307-5p, hsa-miR-5583-5p, hsa-miR-33b-5p, hsa-miR-548f, and hsa-miR-124-3p) whose expression differed between pre- and post-therapeutic patients. Of these, the first three on the list showed high discriminatory power (AUC-ROC = 0.97, 0.96, and 0.93, respectively). In 15 patients with SS, 38 c-miRNAs differentiated post-therapeutic patients from healthy controls, and 18 c-miRNAs were differentially expressed between pre-therapeutic and post-therapeutic samples; none of these dysregulated miRNAs showed high discriminatory power (Table S5). Eleven miRNAs (hsa-miR-376b, hsa-miR-134, hsa-miR-376a-3p, hsa-miR-433, hsa-miR-409-3p, hsa-miR-34a-5p, hsa-miR-136-3p, hsa-miR-31-5p, hsa-miR-136-5p, hsa-miR-329, and hsa-miR-127-3p) were identified during both comparisons.

Similar comparisons in GIST patients identified 60 c-miRNAs that differentiated 12 post-therapeutic patients from healthy controls; of these, three (hsa-miR-30c-5p, hsa-miR-125a-5p, and hsa-miR-194-5p) showed high discriminatory power (AUC-ROC = 0.92-0.9). Twenty-two dysregulated c-miRNAs distinguished pre- from post-therapeutic samples (Table S6). Of these, 14 (hsa-miR-485-3p, hsa-miR-194-5p, hsa-miR-144-3p, hsa-miR-543, hsa-miR-125b-5p, hsa-miR-223-3p, hsa-miR-142-5p, hsa-miR-139-5p, hsa-miR-24-2-5p, hsa-let-7d-3p, hsa-miR-125a-5p, hsa-miR-16-5p, hsa-miR-483-3p, and hsa-miR-641) were common to both comparisons. Among GIST patients, 14 showed disease progression under imatinib treatment. In these patients, pre-therapeutic profiles identified 23 c-miRNAs that were dysregulated when compared with healthy controls; however, 52 dysregulated c-miRNAs were identified in non-progressing patients when compared with controls, and 15 c-miRNAs were shared by both subgroups of GIST patients (Table S6). However, none of the differentially expressed c-miRNAs was identified when the pre-treatment miRNA profiles of patients with further observed progression *vs.* no observed progression under the imatinib treatment were compared.

## Discussion

Expression of miRNA is regulated tightly at the translational level. DNA methylation, histone deacetylation, changes in DNA copy number, and gene mutations affect proteins involved in miRNA processing and maturation. In turn, miRNAs regulate cellular proliferation, differentiation, apoptosis, and metabolism. Therefore, dysregulated miRNA expression may play roles in the pathogenesis of numerous human disorders, including cancer development and progression. Indeed, miRNAs act as functional oncogenes or tumor suppressors; increased expression of oncogenic miRNAs and decreased expression of tumor suppressive miRNAs may contribute to tumorigenesis by promoting cellular proliferation and evasion of apoptosis 46,47. Extracellular miRNAs are highly stable and easily detected in various biofluids, including whole blood, plasma, and serum 25,47. Although circulating miRNAs are considered to be non-invasive biomarkers for cancer diagnosis and prognosis, the association between circulating miRNA profiles and those in tumor tissues remains unclear 26,48. Many studies conducted to date have yielded inconsistent results. Thus, the clinical application of miRNA profiling for early detection of cancer requires further study.

Sarcomas are a group of rare heterogeneous tumors that metastasize predominantly via the blood; this makes them particularly attractive to research involving peripheral blood testing. However, assessment of the prognosis of the vast majority of sarcomas is based only on very simple clinical and pathological factors (e.g., tumor size, grade, and localization), which are clearly inadequate in terms of predicting the prognosis of different sarcoma subtypes 1,4,49.

Several studies have examined c-miRNAs in OS 50, but few studies have been conducted in other sarcoma subtypes. Here, we used next generation sequencing to compare c-miRNA profiles of four sarcoma subtypes: OS, SS, ES, and GIST. We found that most c-miRNAs dysregulated in pre-therapeutic serum samples were sarcoma type-specific; only three were found in all sarcoma types.

To date, miRNA profiling studies have detected between tens and hundreds of dysregulated c-miRNAs in sarcomas (Supplementary Table 2); many of these were identified using high-throughput technologies 25-45. However, we found limited overlap among these findings. Here, we identified only a few c-miRNAs that have been reported by others. We found that 12, 4, 5, and 0 miRNAs had AUC-ROC values > 0.9 in pre-therapeutic samples from EE, GIST, OS, and SS patients, respectively; however, multi-miRNA diagnostic classifiers resulted in higher AUCs for these tumors (1, 0.97, 0.95, and 0.94, respectively).

OS is the most common primary malignant bone tumor. Studies suggest that this tumor is associated with increased levels of circulating miRNAs (including 14q32 miRNAs, miR-95-3p, miR-300, miR-101, miR-542-3p, miR-196a, miR-196b, and miR-491) and with decreased levels of miR-17, miR-497, miR-106a-5p, miR-16-5p, miR-20a-5p, miR-425-5p, miR-451a, miR-25-3p, and miR-139-5p 26-31,47,51,52. Of these, serum miR-199a-5p concentrations are significantly higher in OS patients than in controls, and are significantly lower in post-operative samples than in preoperative samples 53. Most aberrantly expressed c-miRNAs discriminated OS patients from healthy volunteers with high specificity and sensitivity; therefore, they were considered promising diagnostic and prognostic biomarkers for patients with advanced tumors 53. MiR-140 is the first miRNA candidate biomarker associated with drug sensitivity of OS xenografts treated with doxorubicin, cisplatin, and ifosfamide 28.

In addition, TaqMan Low Density Arrays identified 15 miRNAs that are differentially expressed in OS patients compared with healthy controls; of these, miR-215-5p and miR-642a-5p are potential markers for OS diagnosis 32. Genome-wide profiling identified 56 and 164 c-miRNAs that were upregulated and downregulated, respectively, in OS samples. Of these, expression of three miRNAs (miR-21, miR-221, and miR-106a) was particularly high in patient plasma 25. In another study, global profiling of c-miRNAs identified 236 serum miRNAs that were expressed at higher levels in OS patients than in controls 33. The highly dysregulated miR-25-3p correlates with patient prognosis, and might serve as a non-invasive blood-based biomarker for tumor monitoring and prognostic prediction. Correlation between miR-25 expression and bone metabolism was reported previously, with intracellular and extracellular oncogenic functions imputed to miR-25-3p 32,54-56. Another study conducted a meta-analysis to assess the true diagnostic value of circulating miRNAs for early detection of OS 57. The authors concluded that c-miRNAs show great promise for the diagnosis of osteosarcoma in Asian populations, particularly when multiple miRNAs are combined to improve diagnostic accuracy. However, the clinical application of miRNA profiling for early detection of OS requires further study.

ESs are highly aggressive sarcomas characterized by oncogenic chromosomal translocations that lead to formation of fusion genes, mainly the *EWS* gene. Alterations in expression of miRNA in ES involve both EWS/Ets oncogenic fusion-dependent and -independent mechanisms, which contribute to a malignant phenotype. However, some miRNAs with prognostic and therapeutic potential have been identified, including miR-17,92a, miR-106b,25, and miR-106a,363 clusters 34-36,58. It is hypothesized that miR-125b induces chemoresistance in ES/primitive neuroectodermal tumors 59, and that miR-30a-5p interacts with the 3'UTR region of CD99 to regulate its expression 37. Serum miR-125b, one of the most consistently dysregulated miRNAs, may serve as a useful non-invasive biomarker for ES 60; indeed, its expression in ES tumors is lower than that in normal bone tissues 38.

SS shows recurrent alterations in miRNA expression, including that of the miR-143 39 and miR-183 clusters 40. In addition, the miR-17-92 cluster (C13orf25) is upregulated in liposarcoma (the most common STS) 41. Members of the miR-200 family and miR-9/miR-9* are associated with SS and myxoid liposarcomas, respectively. Microarray profiling analysis performed on nine pairs of serum samples obtained from SS patients and healthy individuals identified 49 serum miRNAs that were significantly upregulated in SS patients compared with controls, reaffirming the potential clinical significance of serum miR-92b-3p for identifying SS tumors 42.

GISTs are the most common abdominal sarcoma. They show well-characterized molecular features related to activating mutations in the*KIT* or *PDGFRA* genes. Overexpression of miR-196a is associated with a high-risk grade, metastasis, and poor survival 61; this may allow classification of GIST into various prognostic subtypes 62. Downregulation of miR-221 and miR-222 is implicated in the pathogenesis of GISTs, and may be involved in secondary resistance to tyrosine kinase inhibitors 43,63. Expression of miR-221 and miR-222 is significantly lower in KIT-positive GIST cases, and miR-494 correlates inversely with KIT expression 64,65. Others show that miR-200a-3p correlates negatively with expression of *PDGFRA* 66. Downregulation of miR-320a correlates with shorter time to imatinib resistance in patients with GIST, indicating an important role in the mechanism underlying imatinib resistance in these patients 67. Three other papers show that miRNAs, including miR-218, miR-125a-5p, and miR-518a-5p, play roles regulating sensitivity to imatinib 44,45,68.

The results of the above studies show little overlap: only four miRNAs (hsa-miR-214, hsa-miR-143, hsa-miR-15b, and family hsa-miR-150) are dysregulated in all four tumor types. However, despite the relatively small numbers of samples, particularly in the ES group, we identified ten miRNAs that were dysregulated consistently in at least three tumor types when compared with healthy controls. Eight of these (hsa-miR-124-3p, hsa-miR-96-5p, hsa-miR-582-5p, hsa-miR-450b-5p, hsa-miR-375, hsa-miR-483-5p, hsa-miR-486-5p and miR-150-3p) regulate genes involved in the p53 signaling pathway. A limitation of our study could be a disparity between the ages of GIST and SS patients when compared to control group. The control group was recruited among healthy hospital personnel where young and middle aged individuals dominate. Several studies suggested that the abundance of serum miRNAs can change with age in healthy individuals 69-71, however none of the miRNAs proposed is these studies (Supplementary Table S7) as aging biomarkers differed control from any sarcoma groups.

## Conclusions

We identified aberrant expression of miRNA signatures in serum samples from patients with four different sarcoma subtypes. Differences in miRNA expression profiles between sarcoma patients and healthy volunteers suggest that miRNAs play key roles in sarcoma development. Identification of miRNA patterns unique to individual tumor types may yield new diagnostic biomarkers; as such, miRNAs warrant further investigation as therapeutic targets for sarcoma.

## Supplementary Material

Supplementary figures and tables.

## Figures and Tables

**Table 1:** Patient characteristics

Number of patients	Median age at diagnosis (years)	Gender	Location of the primary tumor	Genetic testing	Best response to imatinib treatment
**Gastrointestinal stromal tumor - unresectable/metastatic**					
**36**	64	Female: 20 Men: 16	Stomach: 21 Small intestine: 6 Duodenum: 5 Esophagus: 1 Rectum: 1 Omentum: 1 Retroperitoneum: 1	*KIT/PDGFRA* mutational status: KIT exon 11: 14 KIT exon 9: 4 PDGFRA: 2 Wild-type: 2 Not assessed: 14	Partial response: 12 Stable disease: 24
**Synovial sarcoma - localized**					
26	44	Female: 15 Male: 11	Upper extremity: 5 Lower extremity: 19 Other: 2	Yes: 16 No: 3 Not assessable: 7	Yes: 9 No: 17
**Ewing sarcoma - locally advanced**					
8	28	Female: 0 Male: 8	Head: 1 Lower limb: 3 Trunk: 4	Yes: 4 No: 2 Not assessable: 2	Yes: 3 No: 5
**Osteosarcoma - localized**					
16	30	Female: 6 Male: 10	Trunk: 2 Upper limb: 3 Lower limb: 10 NA: 1		Yes: 3 No: 12 NA: 1
**Healthy controls**					
30	31	Female:16 Male:14	NA	NA	NA

**Table 2:** Number of c-miRNAs that differentiate between given types of sarcoma

GIST *vs.* OS	GIST *vs.* SS	GIST *vs.* ES	ES *vs.* OS	ES *vs.* SS	SS *vs.* OS
103	28	164	13	23	19

GIST; gastrointestinal stromal tumor, OS; osteosarcoma, SS; synovial sarcoma, ES; Ewing sarcoma

## Abbreviations

c-miRNA: circulating microRNA
STS: soft tissue sarcoma
GIST: gastrointestinal stromal tumor
OS: osteosarcoma
SS: synovial sarcoma
ES: Ewing sarcoma
CT: Computed tomography
ROC-AUC: Receiver operating characteristic curves and area under the curve
